# Genome-Wide Identification and Functional Analysis of the AP2/ERF Transcription Factor Family in Citrus Rootstock under Waterlogging Stress

**DOI:** 10.3390/ijms24108989

**Published:** 2023-05-19

**Authors:** Wen He, Liang Luo, Rui Xie, Jiufeng Chai, Hao Wang, Yan Wang, Qing Chen, Zhiwei Wu, Shaofeng Yang, Mengyao Li, Yuanxiu Lin, Yunting Zhang, Ya Luo, Yong Zhang, Haoru Tang, Xiaorong Wang

**Affiliations:** College of Horticulture, Sichuan Agricultural University, Chengdu 611130, China; hewen0724@gmail.com (W.H.); 2020205010@stu.sicau.edu.cn (L.L.); xierui928@stu.sicau.edu.cn (R.X.); 2021205021@stu.sicau.edu.cn (J.C.); wh2sky@163.com (H.W.); wangyanwxy@sicau.edu.cn (Y.W.); supnovel@gmail.com (Q.C.); 71444@sicau.edu.cn (Z.W.); 71175@sicau.edu.cn (S.Y.); limy@sicau.edu.cn (M.L.); linyx@sicau.edu.cn (Y.L.); asyunting@sicau.edu.cn (Y.Z.); luoya945@sicau.edu.cn (Y.L.); zhyong@sicau.edu.cn (Y.Z.); htang@sicau.edu.cn (H.T.)

**Keywords:** Pujiang Xiangcheng, AP2/ERF, waterlogging, transcriptome, overexpression

## Abstract

Citrus plants are sensitive to waterlogging, and the roots are the first plant organ affected by hypoxic stress. The *AP2/ERF* (APETALA2/ethylene-responsive element binding factors) can modulate plant growth and development. However, the information on *AP2/ERF* genes in citrus rootstock and their involvement in waterlogging conditions is limited. Previously, a rootstock cultivar, *Citrus junos* cv. Pujiang Xiangcheng was found to be highly tolerant to waterlogging stress. In this study, a total of 119 *AP2/ERF* members were identified in the *C. junos* genome. Conserved motif and gene structure analyses indicated the evolutionary conservation of *PjAP2/ERFs*. Syntenic gene analysis revealed 22 collinearity pairs among the 119 *PjAP2/ERFs*. The expression profiles under waterlogging stress showed differential expression of *PjAP2/ERFs*, of which, *PjERF13* was highly expressed in both root and leaf. Furthermore, the heterologous expression of *PjERF13* significantly enhanced the tolerance of transgenic tobacco to waterlogging stress. The overexpression of *PjERF13* decreased the oxidative damage in the transgenic plants by reducing the H_2_O_2_ and MDA contents and increasing the antioxidant enzyme activities in the root and leaf. Overall, the current study provided basic information on the AP2/ERF family in the citrus rootstock and uncovered their potential function in positively regulating the waterlogging stress response.

## 1. Introduction

Citrus plants are the most widely grown and economically important fruit crops worldwide [1]. Citrus production is constantly challenged by various unfavorable environmental conditions. The waterlogging condition is a severe impediment that can trigger a wide range of molecular, physiological, and biochemical changes in plants [2,3]. The citrus plant is cultivated by grafting, which joins the rootstock to the scion [4]. Rootstock is the core source of resistance to various abiotic stresses [5,6,7,8]. However, little is known about the molecular responses to waterlogging stress in citrus, which is crucial for citrus rootstock breeding [5].

The AP2/ERF superfamily plays critical roles in plant growth and development and can be organized into three distinct families (AP2 family, ERF family and RAV family) based on their amino acid sequence similarity and conserved domains. The AP2 family is identified by the presence of two AP2/ERF domains, while the ERF family is characterized by a single AP2/ERF domain. In addition, the RAV family is distinguished by a B3 domain, which is present along with a single AP2/ERF domain [9,10]. Furthermore, the ERF family is categorized into two subfamilies consisting of six groups each. These subfamilies are DREB (dehydration-responsive element binding protein) with groups B1 to B6 and ERF with groups A1 to A6, respectively [9,10]. To date, the ERF family has been extensively identified and characterized in several plants, including strawberry (*Fragaria* × *ananassa*), peach (*Prunus persica*), grape (*Vitis vinifera*), and kiwifruit (*Actinidia valvata*) [11,12,13,14].

Previous studies have shown that the AP2/ERF family plays a pivotal role in enhancing waterlogging tolerance in plants. Specifically, the VII-group has been demonstrated to have particularly significant functions in this regard [15,16]. The overexpression of a group VII ethylene response factor, *ZmEREB180* from *Zea mays* L., enhanced the survival rate, formation of adventitious roots, and regulation of antioxidant levels after long-term waterlogging stress [17]. *TaERFVII1* and *AcERF74/75* also showed that ERF can enhance waterlogging stress tolerance in wheat [18] and in kiwifruit [19], respectively. Although the role of the ERF gene in citrus fruit ripening and cold resistance has been studied [20,21,22,23,24], its function in citrus waterlogging stress remains largely unexplored.

‘Pujiang Xiangcheng’, a novel citrus rootstock cultivar selected from wild *Citrus junos* populations in our previous work [25], exhibits significant resistance to extreme waterlogging after complete acclimatization. We assembled a high-quality genome of the ‘Pujiang Xiangcheng’ de novo, which was sequenced using a combination of Nanopore long-read sequencing, Illumina short reads and chromosome conformation capture (Hi-C) technology. Here, we intended to systematically identify and characterize the AP2/ERF family in ‘Pujiang Xiangcheng’ based on genomic data. Then, the expression patterns of the AP2/ERF members in citrus rootstock under waterlogging stress were analyzed. Finally, we characterized one member, *PjERF13*, by ectopic expression. Our results strengthen the understanding of *PjAP2/ERF* family members and unravels their possible roles in waterlogging adaptation in the citrus rootstock.

## 2. Results

### 2.1. Identification and Characterization of AP2/ERFs in Citrus junos

**Figure 1:**
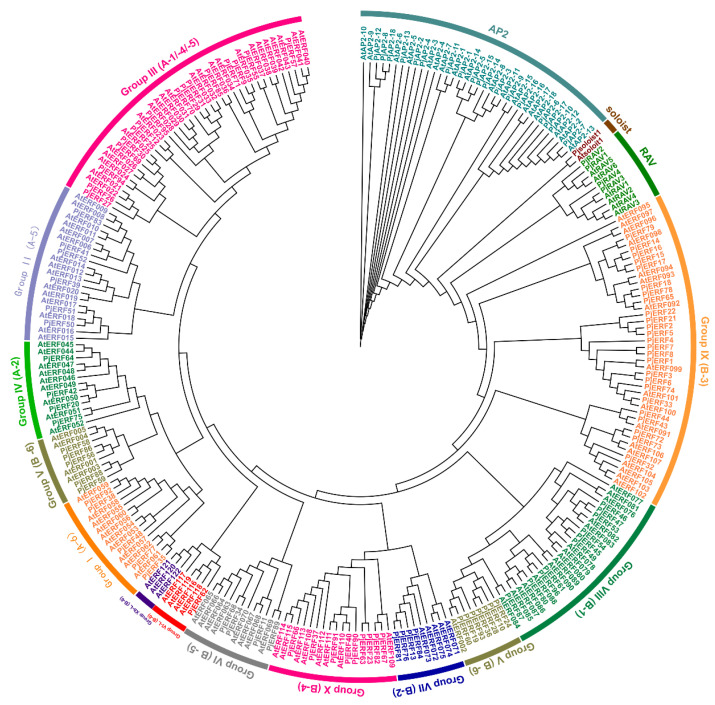
Classification and phylogenetic analysis of PjAP2/ERF family in *Citrus junos*. The PjAP2/ERF protein sequences were clustered into subclades based on the priority classification rule of *Arabidopsis thaliana* AP2/ERFs and were used to construct a maximum likelihood tree with 5000 bootstrap replicates. Bootstrap values greater than 0.7 are indicated by a gray circle in the middle of each branch. Clans and families are indicated using color strips and symbols, respectively. Pj represents *C. junos* Sieb ex Tanaka cv. Pujiang Xiangcheng and At represents *A. thaliana*.

Based on the *C. junos* genome (352.45 Mb, N50 = 3.22 Mb), a total of 119 AP2/ERF superfamily members were identified using a combination of HMMsearch, local Blastp search, and domain verification approaches (Figure 1; Table S1). To analyze the phylogenetic relationships of AP2/ERFs from *C. junos*, an unrooted maximum likelihood (ML) tree was constructed from a trimmed alignment of 119 citrus AP2/ERF protein sequences. Phylogenetic analysis indicated that the AP2/ERFs were categorized into 18 AP2, 4 RAVs, 1 Soloist, and 96 ERFs (I to Xb-L) based on the priority classification rule of *Arabidopsis thaliana* AP2/ERF TFs (Figure 1). We analyzed the ORF (open reading frame) and calculated molecular weight, pI (isoelectric point) and GRAVY (grand average of hydropathy) to obtain additional information on these AP2/ERF proteins. Analysis of physical and chemical properties revealed a wide variation in the characteristics of 119 citrus AP2/ERF proteins, with protein length (number of amino acids) ranging from 67 to 1404, molecular weight ranging from 7.98 to 154.21 kDa, and theoretical isoelectric point ranging from 4.62 to 10.19. The subcellular localization prediction showed that most of the citrus AP2/ERF proteins (106 of 119) were localized in the nucleus, followed by the chloroplast (11 of 119) (Table S1).

### 2.2. Gene Structure and Conserved Motif Analyses of PjAP2/ERFs

**Figure 2:**
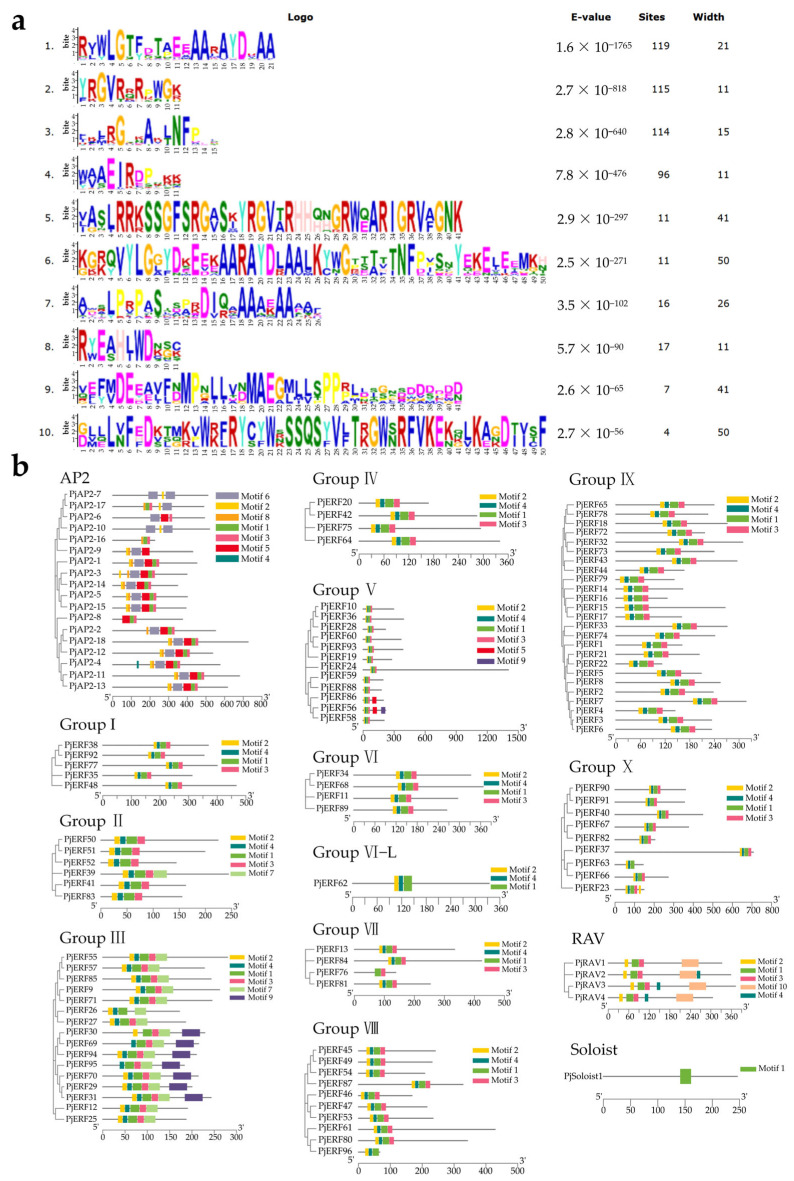
The conserved motif analysis of 119 *PjAP2/ERFs* based on a comprehensive phylogenetic tree. (**a**) The amino acid sequences of motifs, (**b**) Analysis of ten conserved motifs of *PjAP2/ERFs* according to the phylogenetic relationships.

Proteins belonging to the same group are likely to share similar conserved motifs [9]. To explore the structural characteristics of the *PjAP2/ERF* genes, the intron/exon arrangement of each *PjAP2/ERF* was analyzed (Figure S1). More than half of the *PjAP2/ERFs* were intronless, and the remaining *PjAP2/ERF* coding regions were interrupted by one or more introns of varying sizes. Furthermore, a search for shared conserved motifs in *PjAP2/ERFs* subfamily predicted ten motifs using MEME, which were named motifs 1 to 10 (Figure 2a). The motifs 1, 2, 3, and 4 were distributed in almost all *PjAP2/ERFs*, while other motifs were distributed among various subgroups. In particular, the ERF/DREB subgroup members contained Motif 7; RAV subfamily TFs contained Motif 9; AP2 TFs contained four motifs, Motif 5, 6, 8, and 10 (Figure 2b).

### 2.3. Chromosomal Location and Synteny Analysis of PjERFs

**Figure 3:**
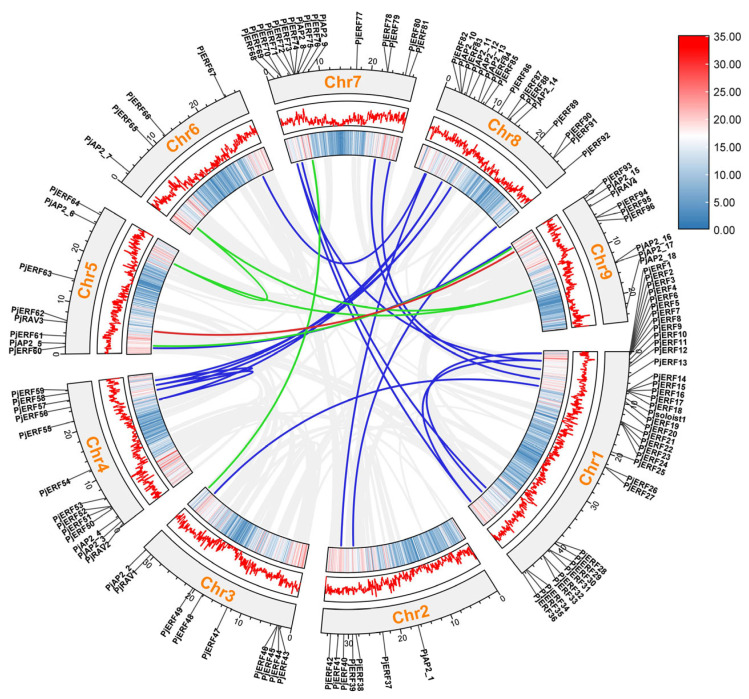
Collinearity analysis of AP2/ERF transcription factor of *C. junos* Sieb. Ex Tanaka cv. Pujiang Xiangcheng. The blue and red colors and the broken line in the boxes represent gene density, which refers to the total number of genes contained in 100,000 base pairs. The blue collinear lines within the circle represent the ERF family, the red lines represent the RAV family, and the green lines represent the AP2 family.

To determine the genomic distribution of *PjAP2/ERF* genes, their position on each chromosome was investigated. The 119 *PjAP2/ERF* genes were unevenly distributed among the nine chromosomes (Figure S2). The maximum number of *PjAP2/ERF* genes (37) were located on chromosome 1, while chromosome 6 had the smallest number of *PjAP2/ERFs* (4). Subsequently, a collinearity analysis was conducted to investigate the homologous genes and their evolutionary relationship, as shown in Figure 3, 22 collinearity pairs were detected, including 5 pairs of AP2 transcription factors, 16 pairs of ERF/DREB transcription factors, and 1 pair of RAV transcription factors. Among the five pairs of collinear AP2 transcription factors, *PjAP2-6*, *PjAP2-7*, and *PjAP2-16* exhibited a collinear relationship. In addition, among the ERF/DREB transcription factors, *PjERF55*, *PjERF57*, and *PjERF85* showed a collinear relationship, while *PjERF56*, *PjERF58*, and *PjERF86* had a collinear relationship. Further, *PjERF68* showed collinearity with *PjERF11* and *PjERF34*.

### 2.4. The Expression of PjAP2/ERFs in Response to Waterlogging Stress

**Figure 4:**
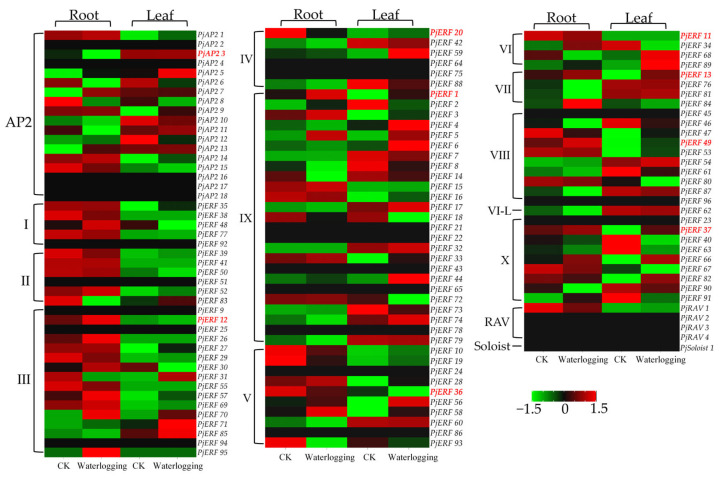
Waterlogging responsiveness and expression profiles of *PjAP2/ERFs* in root and leaf. Heat map showing expression of the *PjAP2/ERF* genes based on log_10_^(FPKM + 0.01)^ values. Red font represents differential expression AP2/ERF in root or leaf. CK: for control treatments, plants were watered every three days for 35 days. Waterlogging: water was kept at the same level for the entire 35-day duration.

To explore the role of *PjAP2/ERF* in plant tolerance to waterlogging stress, their expression profiles were evaluated in leaf and root samples (waterlogging stress after processing treatment for 35 days and controls, China National GeneBank DataBase under the project: CNP0004172). As shown in Figure 4, some *PjAP2/ERFs* were not expressed in the root and leaf. Three DEGs (*PjERF36* were downregulated, *PjERF37* was upregulated) were identified in the leaf, while six DEGs (*PjAP2 3* and *PjERF20* were downregulated, *PjERF1*, *PjERF11, PjERF12,* and *PjERF49* were upregulated) were detected in the root. Previous studies have shown that the *ERF*-*VII* group plays a particularly important role in improving plant waterlogging tolerance [15,16]. In this study, the expression level of *PjERF13,* from the *ERF*-*VII* group, increased significantly after 35 days of waterlogging in both root and leaf, which also showed differential expression in previous research under waterlogging stress in citrus roots [26]. These results suggested that *PjERF13* was significantly induced by waterlogging and might play an essential role in the waterlogging stress response in citrus.

### 2.5. Validating the Expression of PjERFs during Waterlogging Conditions

**Figure 5:**
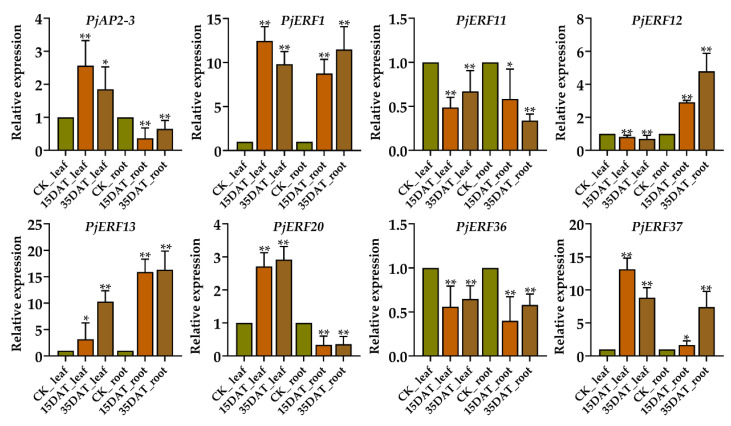
Expression levels of eight *PjAP2/ERFs* in the leaf and root of ‘Pujiang Xiangcheng’ under waterlogging stress. The data represent the means ± standard deviation (SD), ** *p* < 0.01, * *p* < 0.05, Tukey’s test. All significances were data from 15 DAT (days after treatment) and 35 DAT compared to the control, respectively.

To further validate the differential expression of eight *PjAP2/ERFs* in response to waterlogging stress in *C. junos*, qRT-PCR analysis was carried out in three rootstock genotypes with different tolerance abilities to waterlogging (Figure 5). Consistent with the transcriptome data, the expression levels of *PjERF1* and *PjERF12* were increased in the root of ‘Pujiang Xiangcheng’ under waterlogging stress compared to the control. The *PjERF13* expression was significantly upregulated by waterlogging in both leaf and root. The expression of *PjERF37* was induced by waterlogging in the leaf. In addition, we observed that *PjERF1*, *PjERF20*, and *PjERF23* were upregulated in the ‘Pujiang Xiangcheng’ leaf under waterlogging stress, while the transcript level of *PjERF37* was increased in the root. Overall, the qRT-PCR results were consistent with the RNA-seq data, indicating that the expression of these genes was influenced by waterlogging stress in ‘Pujiang Xiangcheng’.

### 2.6. Subcellular Localization of PjERF13

**Figure 6:**
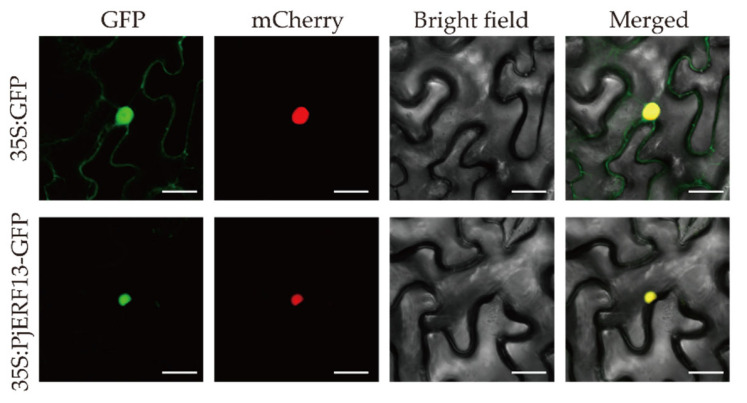
PjERF13 is localized to the nucleus. The fusion construct (35S:PjERF13::GFP) or an empty vector (35S:GFP) was co-transformed with a nuclear marker gene *VirD2NLS* fused to mCherry in tobacco (*Nicotiana benthamiana*) leaves. Confocal microscopic images of epidermal cells were captured under a bright field microscope. The yellow color is merged GFP and mCherry signals, while the red is for mCherry fluorescence signals. Scale bars = 25 µm.

Based on previous findings on waterlogging conditions in citrus rootstocks [26], *PjERF13* from the *ERF*-*VII* group was selected for further study to validate its function and responsiveness to stress. First, we performed subcellular localization to identify the transcription factor features of the *PjERF13* genes. We cloned coding sequences of the PjERF13 protein for plasmid constructs encoding a fusion protein containing PjERF13 protein and green fluorescent protein (PjERF13::GFP) driven by the 35S promoter. The empty vector harboring GFP and mCherry used as positive controls showed a diffused distribution of the green and red fluorescence signals in the entire cells and nucleus, respectively. The GFP fluorescence signals of *PjERF13*::GFP fusion proteins were predominantly localized in the nucleus (Figure 6). The subcellular localization results indicated that *PjERF13* is a nuclear-localized protein.

### 2.7. Overexpression of PjERF13 Enhanced the Tolerance of Tobacco to Waterlogging Stress

**Figure 7:**
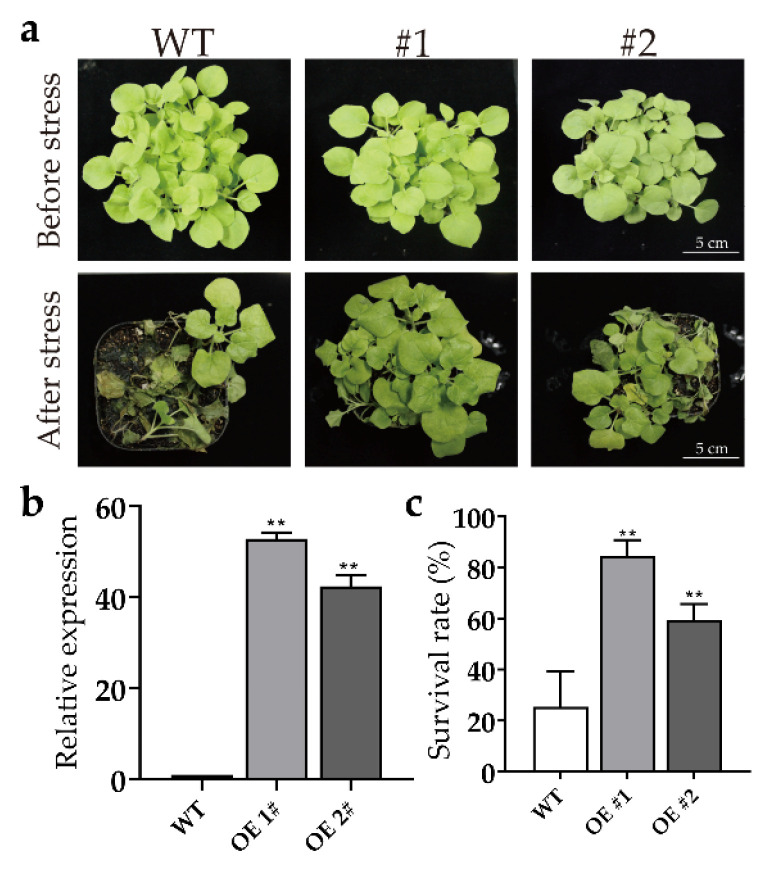
Overexpression of *PjERF13* in tobacco. (**a**) the phenotype of *PjERF13*-overexpressing and WT plants under waterlogging stress. (**b**) the relative expression of *PjERF13* in transgenic tobacco lines detected by qRT-PCR. (**c**) the survival rate of transgenic plants compared to the WT. WT indicates the wild-type tobacco; #1 and #2 represent the two independent transgenic lines overexpressing *PjERF13.* The data represent the means ± standard deviation (SD), ** *p* < 0.01, Tukey’s test.

Based on our data and previous research findings [26], *PjERF13* may play an important role in the *C. junos* seedlings under waterlogging stress. The heterologous overexpression of *PjERF13* greatly enhanced the tolerance of tobacco to waterlogging stress (Figure 7a). The relative expression level of *PjERF13* was significantly increased more than 40 times in the transgenic plants compared to the WT (wild-type tobacco) (Figure 7b), suggesting its successful overexpression in tobacco. In addition, under the waterlogging conditions, the transgenic tobacco plants exhibited up to 80% survival rate, while the WT displayed only a 20% survival rate, which is four times lower than that in *PjERF13*-overexpressing plants (Figure 7c). These results demonstrated that *PjERF13* might positively regulate the citrus resistance to waterlogging stress.

### 2.8. Overexpression of PjERF13 Decreased the Oxidative Damage in the Root

**Figure 8:**
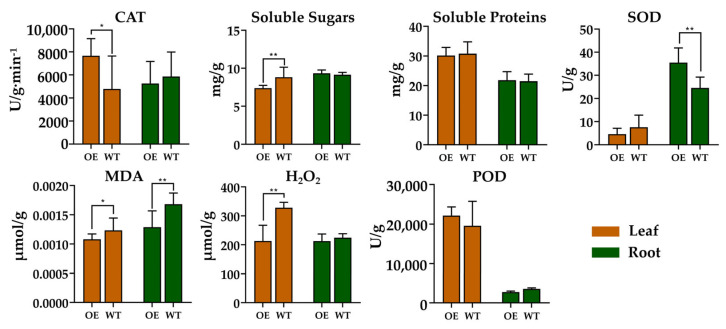
The changes in physiological oxidative traits in the root and leaf of *PjERF13*-overexpressing transgenic tobacco lines compared to the WT under waterlogging stress. OE represents plants overexpressing *PjERF13*, while WT indicates the wild type. The data represent the means ± standard deviation (SD), ** *p* < 0.01, * *p* < 0.05, Tukey’s test.

To detect the impact of *PjERF13* on physiological oxidative characteristics, related traits were further estimated in the *PjERF13*-overexpressing tobacco plants (Figure 8). The results showed that the SOD activity was significantly increased in root, while the MDA content was reduced mainly in the plant leaves and roots in plants overexpressing *PjERF13* compared to the WT. Furthermore, the activities or levels of POD, CAT, H_2_O_2_, and soluble sugars and proteins content in root were not altered by the overexpression of *PjERF13.* The CAT activity was greatly enhanced, while the soluble sugars, MDA, and H_2_O_2_ contents were decreased in the leaf of *PjERF13* overexpressing plants. These results indicated a lower oxidative damage level in the *PjERF13*-overexpressing plants under waterlogging stress compared to the WT.

## 3. Discussion

The AP2/ERF gene family is widely distributed in plants as transcription factors regulating multiple biological processes [9,15]. Earlier, ‘Pujiang Xiangcheng’ from *C. junos* was screened as a waterlogging-tolerant genotype. Recently, a high-quality chromosome-scale genome of *C. junos* cv. Pujiang Xiangcheng was assembled de novo (352.45 Mb, N50 = 3.22 Mb)*,* and the transcriptome analysis was performed (China National GeneBank DataBase under the project CNP0004172). In the current study, we reported 119 *ERF* genes for the first time in *C. junos* based on the genomic data, similar to the known number of ERFs in the *C. sinensis* cv. ‘Newhall’ (126) [27]. To further analyze the relationship among the *AP2/ERF* gene family in citrus, we explored them using the published genome data of various *Citrus* species (downloaded from Citrus Pan-genome to Breeding Database, http://citrus.hzau.edu.cn (accessed on 10 October 2022)), including *C. clementina*, *C. sinensis*, *C. grandis*, *C. ichangensis*, *P. trifoliata*, *Fortunella hindsii*, *C. reticulata*, *C. medica*, and *Atalantia buxfoliata* (Table S2). Overall, the number of AP2/ERF transcription factors in ‘Pujiang XiangCheng’ (119) was relatively small among the compared citrus species, except for *C. ichangensis* and *Atalantia buxfoliata*. However, the proportion of each subgroup and group was relatively consistent with other *Citrus* species, indicating the conservation of this TF family to some extent during the process of maturation.

Phylogenetic analysis classified eleven groups of AP2/ERF proteins in ‘Pujiang Xiangcheng’. There was no difference between PjAP2/ERF proteins and AtAP2/ERF proteins (Figure 1), which implies that the identification and classification results of *PjAP2/ERF* are accurate and reliable. ERF genes classified in the same group usually perform similar functions [10]. For instance, four genes of the A1 subgroup in *Arabidopsis*, *AtCBF1* to *AtCBF4*, were known to play crucial roles in cold and drought stress responses [28,29], which also exhibit resistance to cold stress in cucumber (*Cucumis sativus* L.) [30]. However, only a few studies are available on the ERF family in *Citrus*. ERF genes from sweet orange and Clementine have been identified and were shown to play a role in peel degreening in oranges [21,27]. ERFs in *P. trifoliata* exhibited their role in cold tolerance [22,23,24]. Previous studies on the waterlogging stress of citrus have found three ERFs were associated with hypoxia stress [26]. In this study, three differentially expressed ERF genes were identified in the leaf, while six differentially expressed ERF genes were detected in the roots. Due to limited available data on these ERFs, their function in waterlogging tolerance needs to be intensively dissected.

The positive effects of ERF-VII members on resistance to waterlogging stress have been reported in many plants, including *Arabidopsis* [31,32], *Mentha* [33], rice [34], and *Actinidia deliciose* [35]. Five members of the *AtERF-VII* group, including *AtERF71-75,* have been demonstrated to play crucial roles in waterlogging and hypoxia stress response [36,37]. We found that four members of group *VII*, including *PjERF13,* were strongly induced under waterlogging stress. Therefore, it is imperative to further study the function of *PjERFs* under waterlogging conditions. However, studies on the ERF gene family in citrus rootstock are scarce. In rice and *Arabidopsis*, *Sub1A* and two hypoxia responsive ERF genes (*HRE1* and *HRE2*), flooding-induced ERFs play a crucial role in submergence tolerance [31,38]. In the current study, the transcript level of PjERF13 was found to be significantly upregulated in 35S:PjERF13 transgenic plants after waterlogging treatment, which belong to the same ERF group. PjERF13-overexpressing plants showed a lower oxidative damage level under waterlogging stress compared to the WT, including the soluble sugars, MDA, and H_2_O_2_ contents were decreased in the leaf and the MDA content was decreased in the root. These results provided a foundation for further elucidating the role of *PjAP2/ERFs* in waterlogging stress resistance and its underlying mechanism.

## 4. Materials and Methods

### 4.1. Identification and Sequence Analysis of AP2/ERF Genes in Citrus junos

Putative ERF sequences were retrieved based on the genome data of *C. junos* Sieb. Ex Tanaka cv. Pujiang Xiangcheng (352.45 Mb, N50 = 3.22 Mb). The genome and proteome data of *A. buxfoliata*, *C. ichangensis*, *C. medica*, *C. reticulata*, *C. grandis*, *C. clementina*, *C. sinensis*, *F. hindsii*, and *P. trifoliata* were downloaded from CPBD (Citrus Pan-genome to Breeding Database, http://citrus.hzau.edu.cn (accessed on 10 October 2022)). All the proteomes of the ten plants were scanned by our local server HMMER3.1 (AP2-specific hidden Markov Model profile, PF00847, http://pfam.xfam.org (accessed on 1 September 2022)) and web tool pfam 35.0 (http://pfam.xfam.org/ (accessed on 1 September 2022)) in batch mode with an E value of 0.01. The verified sequences were recognized as candidate ERF family members in *C. junos*. The ExPASy-ProtParam online website (http://web.expasy.org/protparam/ (accessed on 1 October 2022)) was used to evaluate the basic physicochemical properties of the PjAP2/ERF proteins, including amino acid length, theoretical pI, molecular weight and grand average of hydropathicity (GRAVY). Subcellular location was analyzed by ProtComp v9.0 (http://linux1.softberry.com/berry.phtml?topic=protcomppl&group=programs&subgroup=proloc (accessed on 15 October 2022)).

### 4.2. Phylogenetic Analysis, Chromosomal Distribution, and Conserved Motif Identification

The *Arabidopsis AP2/ERFs* sequences were retrieved from TAIR (The Arabidopsis Information Resource, http://www.arabidopsis.org/index.jsp (accessed on 15 October 2022)) database to conduct a local Blast search. The multiple sequence alignment of all AP2/ERF protein sequences encoding the conserved AP2 domain was constructed using MUSCLE. A phylogenetic tree of *AP2/ERFs* from *C. junos* and *Arabidopsis* was constructed using the neighbor-joining method in MEGA-X with 5000 bootstrap replicates and the Poisson model [39]. Subsequently, the putative *PjAP2/ERF* subfamilies were classified into different groups. The physical locations of *PjAP2/ERFs* on the *C. junos* genome were extracted from the genome annotation.

### 4.3. Plant Growth and Waterlogging Stress Treatments

Germinating tubers of ‘Pujiang Xiangcheng’ were grown in pot experiments. After three months of growth, seedlings with the same height and phenotype were used for the following experiments. The plastic buckets were filled with nutrient solution 1 cm above the junction of stem and root and secured by a sponge. On day 15 and 35 of the experiment (Figure S3), the seedlings were carefully washed with double-distilled water. Subsequently, shoots and roots were separately harvested, quickly frozen using liquid nitrogen and stored at −80 °C.

### 4.4. Analysis of PjAP2/ERFs Expression under Waterlogging Stress

An equal number of leaves from five seedlings were pooled together as one replicate. Total RNA was extracted from the leaf and root tissues of Pj and purified using the EasyPure^®^ Plant RNA Kit (TransGen Biotech Co., Ltd., Beijing, China), as per the manufacturer’s protocol. Three independent replicates were collected for RNA extraction, as described previously [40]. Clean reads were downloaded from the China National GeneBank DataBase (CNGBdb) database sequence read archive under the accession number CNP0004172. Before sequence assembly, the adapter sequences and low-quality reads were removed from the raw data. TopHat2 (v.2.1.1) [41] was used to map clean reads to the reference genome of *C. junos* (352.45 Mb, N50 = 3.22 Mb). The number of fragments per kilobase of transcripts per million mapped reads (FPKM) was calculated using the RSEM tool (v.1.3.3) [42]. The average FPKM values of three replicates were calculated as the expression level of genes in each sample.

### 4.5. Real-Time PCR Analysis

To further validate the transcriptome data, eight *AP2/ERF* genes were selected for qRT-PCR. The primers were designed using Primer Premier 5 software (Premier Biosoft International, Palo Alto, CA, USA) and listed in Table S3. The qRT-PCR was performed in a 10 µL reaction volume using the TransStart^®^ Green qPCR SuperMix (TransGen Biotech Co., Ltd., Beijing, China) on a CFX96 Touch^TM^ Real-Time PCR detection system (Bio-Rad, Hercules, CA, USA). The PCR program was performed as described previously [40]. The 2^−∆∆Ct^ method was used to calculate the relative expression of genes. The *EF-1α* gene was used as an internal control [43].

### 4.6. Subcellular Localization Analysis of PjERF13

The empty vector was linearized using double enzyme digestion. For the subcellular localization vector, XbaI and BamHI enzymes were used. The sequence of *PjERF13* was amplified and inserted into pYTSL-16 (modified from pMDC83-35S and pSITE-2NB, [44]) by using the CloneEx-press II One Step Cloning Kit (Vazyme, Nanjing, China). We constructed the plasmid encoding a fusion protein of PjERF13 and green fluorescent protein (PjERF13::GFP) driven by the 35S promoter. The primers were designed by Primer Premier 5 (Table S3, PjERF13-SL-F and PjERF13-SL-R). The plasmid was further transformed into *Agrobacterium tumefaciens* strain GV3101. The empty vector was used as a control. The constructs were transiently expressed in tobacco leaves for subcellular localization analysis.

### 4.7. Expression Pattern of PjERF13 Genes under Waterlogging Stress

Tobacco (*Nicotiana benthamiana*) was used for transient genetic transformation and stress treatments. The plants were cultured in a light incubator at 28 °C with 16 h light and 20 °C with 8 h dark cycle conditions. The sequence of PjERF13 was amplified and inserted into the restriction enzyme site of pCambia2301 by using CloneEx-press II One Step Cloning Kit (Vazyme, Nanjing, China). The primers were designed by Primer Premier 5 (Table S3, PjERF13-2301-F and PjERF13-2301-R). The plasmid construction of pCAMBIA2301::35S::PjERF13 was transformed into GV3101 and subsequently transformed into tobacco. The 4-week-old overexpressing tobacco seedlings were treated with waterlogging stress for 2 weeks. Three biological replicates were collected, containing five plants in each replicate. The antioxidant enzyme activities, including SOD (EC 1.15.1.1), POD (EC 1.11.1.7), and CAT (EC 1.11.1.6), were determined as described previously [45]. Soluble sugar and protein contents were analyzed in the leaves using the previously described method [46]. The hydrogen peroxide (H_2_O_2_) and malondialdehyde (MDA) contents were determined using a spectrophotofluorometer [47].

### 4.8. Statistical Analysis

All data were entered into Microsoft Excel 2020 for collation and calculation. Significant differences between grafted combinations were analyzed by Tukey’s method. Column bar and box and whisker plots were generated using GraphPad Prism (v. 7.04). The data of three independent biological tests were reported as the means ± standard deviation (SD).

## 5. Conclusions

In the present study, we identified 119 *PjAP2/ERF* genes from the genomic data of *C. junos* and analyzed their evolutionary relationship, conserved motifs, and expression profiling, which helped in classifying these genes and understanding their responses to waterlogging stress. Eight candidate genes were obtained by qRT-PCR analysis. The present study helps to further understand the role of ERF in the tolerance of *C. junos* to waterlogging stress and provides the basis for further studying ERF genes involved in waterlogging stress responses in citrus. Moreover, one candidate gene *PjERF13* was obtained, which provides a reliable direction for further research on the molecular mechanism of citrus rootstock waterlogging tolerance.

## Data Availability

The datasets presented in this study can be found in the China National GeneBank DataBase (CNGBdb) under accession number CNP0004172.

## Supplementary Materials

The following supporting information can be downloaded at: https://www.mdpi.com/article/10.3390/ijms24108989/s1.
