# Supplementary material for: Genome-Wide Identification and Functional Analysis of the AP2/ERF Transcription Factor Family in Citrus Rootstock under Waterlogging Stress
Source: Int J Mol Sci. 2023 May 19;24(10):8989. doi: 10.3390/ijms24108989 (PMC10218916; doi:10.3390/ijms24108989)
Supplement: Supplementary file 1 [file ijms-24-08989-s001.zip › Supplementary Figure.pdf]

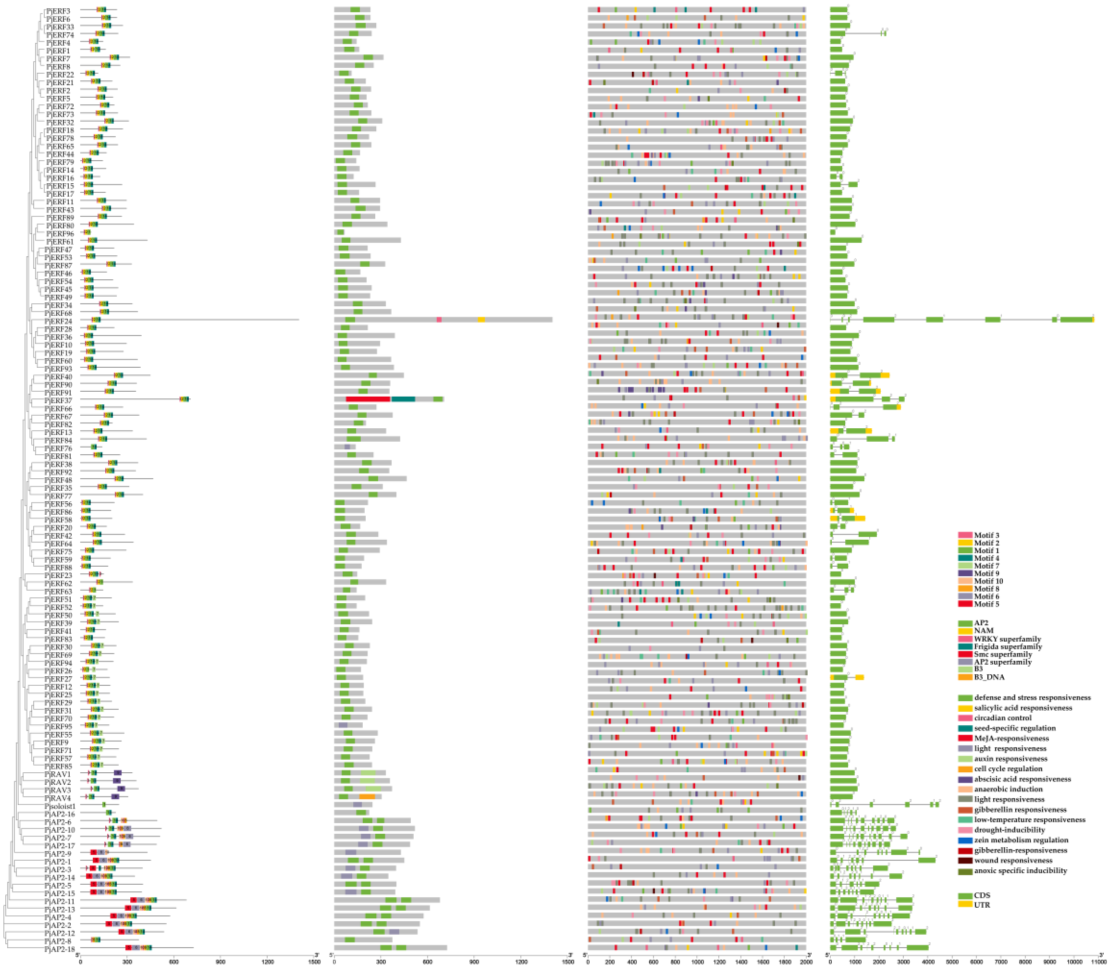

Figure S1. Phylogenetic relationship, conserved protein motif and gene structure analysis of *PjAP2/ERFs*.

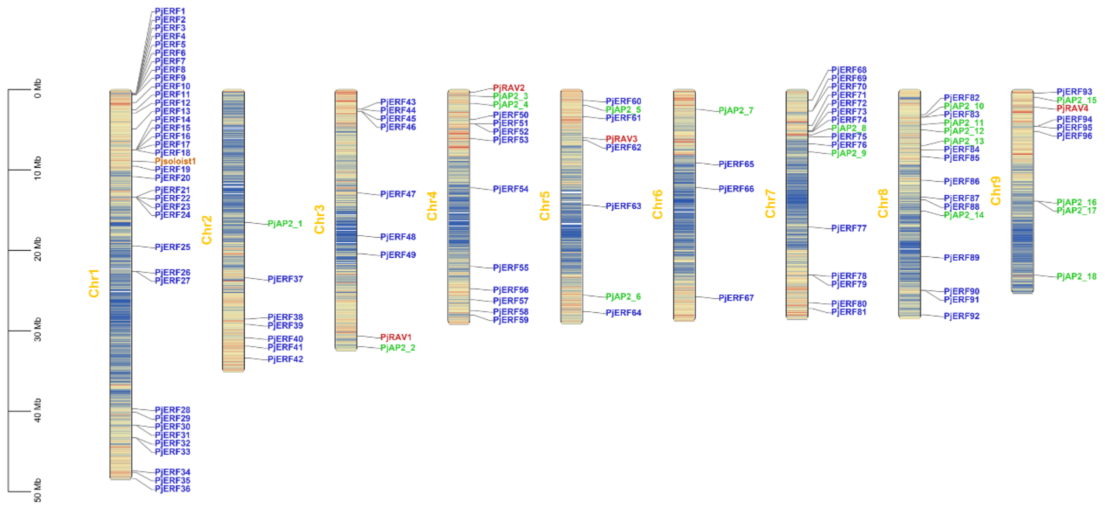

Figure S2. The distribution of *Citrus junos AP2/ERF* genes in 9 chromosomes. The scale bar on the left shows the length of the chromosome (Mb) and the chromosome number is displayed at the left of each chromosome.

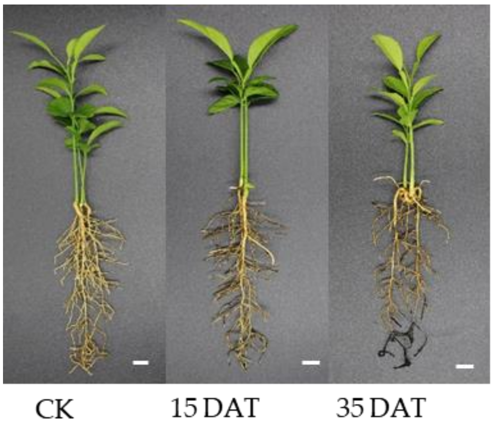

Figure S3. Physiological analysis of *Citrus junos* cv. Pujiang Xiangcheng seedlings in response to waterlogging stress. Bars = 1 cm. DAT: Days After Treatment.
